# 1032. Are enterococcal blood stream infections an independent risk factor for a poorer 5-year survival or just a marker for severity of illness? - The Munich Multi-Centric Enterococci cohort

**DOI:** 10.1093/ofid/ofad500.063

**Published:** 2023-11-27

**Authors:** Tobias Bachfischer, Milena Wurst, Siranush Karapetyan, Alexander Hapfelmeier, Jochen Schneider, Sabine Gleich, Karl Dichtl, Roland Schmid, Laura Wagner, Julian Triebelhorn, Johanna Erber, Ulrich Seybold, rainer burgkart, Andreas obermeier, Floran Voit, Dirk Busch, patrick Rämer, Christoph Spinner, kathrin Rothe

**Affiliations:** Department of Internal Medicine II, University Hospital rechts der Isar, Technical University of Munich, School of Medicine, Munich, Germany, Munich, Bayern, Germany; Department of Internal Medicine II, University Hospital rechts der Isar, Technical University of Munich, School of Medicine, Munich, Germany, Munich, Bayern, Germany; Institute of AI and Informatics in Medicine, University Hospital rechts der Isar, School of Medicine, Technical University of Munich, Munich, Germany, Munich, Bayern, Germany; Institute of AI and Informatics in Medicine, University Hospital rechts der Isar, School of Medicine, Technical University of Munich, Munich, Germany, Munich, Bayern, Germany; Department of Internal Medicine II, University Hospital rechts der Isar, Technical University of Munich, School of Medicine, Munich, Germany, Munich, Bayern, Germany; Public Health Service, City of Munich, Munich, Germany., Munich, Bayern, Germany; Max von Pettenkofer-Institut für Hygiene und Medizinische Mikrobiologie, Munich, Bayern, Germany; Department of Internal Medicine II, University Hospital rechts der Isar, Technical University of Munich, School of Medicine, Munich, Germany, Munich, Bayern, Germany; Department of Internal Medicine II, University Hospital rechts der Isar, Technical University of Munich, School of Medicine, Munich, Germany, Munich, Bayern, Germany; Department of Internal Medicine II, University Hospital rechts der Isar, Technical University of Munich, School of Medicine, Munich, Germany, Munich, Bayern, Germany; Technical University of Munich, School of Medicine – University Hospital, Department of Internal Medicine, Gastroenterology, Munich, Bayern, Germany; Department of Medicine IV, Hospital of the LMU Munich, Munich, Bayern, Germany; Clinic of Orthopaedics and Sports Orthopaedics, University Hospital rechts der Isar, Technical University of Munich, School of Medicine, Munich, Germany, Munich, Bayern, Germany; Clinic of Orthopaedics and Sports Orthopaedics, University Hospital rechts der Isar, Technical University of Munich, School of Medicine, Munich, Germany, Munich, Bayern, Germany; Department of Internal Medicine II, University Hospital rechts der Isar, Technical University of Munich, School of Medicine, Munich, Germany, Munich, Bayern, Germany; Institute for Medical Microbiology, Immunology and Hygiene, University Hospital rechts der Isar, Technical University of Munich, School of Medicine, Munich, Germany, Munich, Bayern, Germany; Department of Hospital Hygiene and Infection Control, Munich Municipal Hospital Group, Munich, Germany, Munich, Bayern, Germany; Department of Medicine II, University Hospital rechts der Isar, Technical University of Munich, Munich, Germany, Munich, Bayern, Germany; Institute for Medical Microbiology, Immunology and Hygiene, University Hospital rechts der Isar, Technical University of Munich, School of Medicine, Munich, Germany, Munich, Bayern, Germany

## Abstract

**Background:**

To assess the long-term survival of patients with enterococcal bloodstream infections (BSI), encompassing various species and resistance patterns, in comparison to Escherichia coli (*E. coli) BSI*.

Recruitment process

**Figure:**
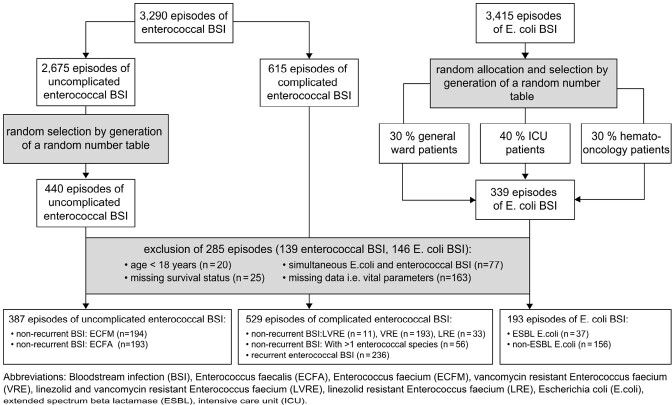

Graphical Abstract

**Figure:**
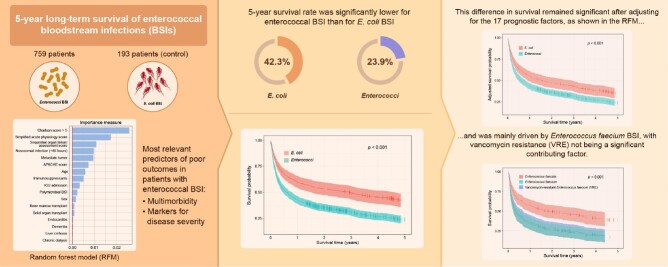

**Methods:**

Between 2010 and 2019, 3,290 enterococcal and 3,415 E. coli BSI were retrospectively screened in seven hospitals in Munich, Germany. All vancomycin (VRE), vancomycin/linezolid (LVRE), and linezolid (LRE) resistant *Enterococcus faecium* (ECFM) BSI were included. *Enterococcus faecalis* (ECFA), vancomycin/Linezolid-susceptible ECFM, and *E. coli* BSI were randomly assigned. Cox-regression models were used to assess survival as the primary endpoint and were adjusted for limiting prognostic factors, which were measured for their importance using a random forest model (RFM).

**Results:**

952 patients with 916 episodes of enterococcal BSI and 193 E. coli BSI episodes were analysed. RFM identified multimorbidity and markers for disease severity as most indicative of low survival in enterococcal BSI. The 5-year survival was significantly lower for enterococcal BSI than for *E. coli* BSI (23.9% vs. 42.3%; p< 0.001). This difference remained significant in the Cox-regression analysis after adjusting for 17 prognostic factors and excluding patients with limited life expectancy (metastatic tumour disease, Charlson comorbidity index ≥5). Adjusted 5-year survival between *E. coli* and ECFA was similar but significantly different between ECFA and ECFM BSI (29.2% vs. 21.7%; p=0.002). The analysis conducted on monomicrobial ECFM and VRE BSI indicated that their respective 5-year survival was similar (19.6% vs. 21.2%; p=0.753).

**Conclusion:**

ECFM BSI seems to be an independent risk factor for poor long-term survival. However, additional vancomycin resistance does not appear to be a contributing factor.

**Disclosures:**

**Jochen Schneider, MD**, JS reports grants, personal fees, and nonfinancial support from AbbVie, Gilead Sciences, Janssen-Cilag, GSK/ViiV Healthcare, and MSD, Dr. Falk Pharma: Grant/Research Support **Karl Dichtl, MD**, KD reports grants from Euroimmun Medizinische Labordiagnostika and Fujifilm Wako Chemicals Europe outside of this study.: Advisor/Consultant **Ulrich Seybold, MD**, reports personal fees and nonfinancial support from Gilead Sciences, ViiV Healthcare, Janssen-Cilag, Falk Foundation, Sanofi-Aventis.: Advisor/Consultant **Christoph Spinner, MD**, AbbVie, Gilead Sciences, Janssen-C,MSD, Cepheid, GSK, ViiV Healthcare, AstraZeneca, Apeiron, Braun, Pfizer, Novartis, Lilly, Biontech…: Advisor/Consultant|AbbVie, Gilead Sciences, Janssen-C,MSD, Cepheid, GSK, ViiV Healthcare, AstraZeneca, Apeiron, Braun, Pfizer, Novartis, Lilly, Biontech…: Board Member|AbbVie, Gilead Sciences, Janssen-C,MSD, Cepheid, GSK, ViiV Healthcare, AstraZeneca, Apeiron, Braun, Pfizer, Novartis, Lilly, Biontech…: Grant/Research Support|CDS reports grants and personal fees from AbbVie, grants, fees and non-financial support from Gilead Sciences, grants and personal fees from Janssen-C: Advisor/Consultant|CDS reports grants and personal fees from AbbVie, grants, fees and non-financial support from Gilead Sciences, grants and personal fees from Janssen-C: Board Member|CDS reports grants and personal fees from AbbVie, grants, fees and non-financial support from Gilead Sciences, grants and personal fees from Janssen-C: Grant/Research Support

